# The possible molecular mechanisms of bisphenol A action on porcine early embryonic development

**DOI:** 10.1038/s41598-017-09282-2

**Published:** 2017-08-17

**Authors:** Jing Guo, Ming-Hui Zhao, Kyung-Tae Shin, Ying-Jie Niu, Yong-Dae Ahn, Nam-Hyung Kim, Xiang-Shun Cui

**Affiliations:** 1 Department of Animal Sciences, Chungbuk National University, Cheongju, Chungbuk 361-763 Republic of Korea; 2 State Key Laboratory of Veterinary Biotechnology, Heilongjiang Provincial Key Laboratory of Laboratory Animal and Comparative Medicine, Harbin Veterinary Research Insititute of Chinese Academy of Agricultural Sciences, Harbin, 150069 China; 3 Cheongwon Natural Island, 203, Urongni, Seowongu, Cheongju, Chungbuk 362-823 Republic of Korea

## Abstract

Bisphenol A (BPA) is an environmental contaminant widely used in the plastic industry. BPA has been demonstrated to be an endocrine disruptor and has an adverse effect on the embryonic development of mammals. However, the mechanism of action of BPA is limited. In this study, we investigated the role and mechanism of BPA in porcine embryonic development. First, the parthenotes were treated with different concentrations of BPA. We found that blastocyst formation was impaired and the parthenotes were arrested at the 4-cell stage after treatment with 100 μm BPA. Second, ROS increased following the addition of BPA, which further caused mitochondrial damage, and cytochrome c was released from the mitochondria to induce apoptosis. The adaptive response was demonstrated through LC3 immunofluorescence staining and by assessing autophagy-related gene expression. In addition, BPA caused DNA damage through the p53-p21 signaling pathway. Thus, our results indicate that BPA displays an adverse effect on porcine early embryonic development through mitochondrial and DNA damage.

## Introduction

Bisphenol A (BPA) is an environmental contaminant widely used in the plastic industry for the manufacture of bottles, containers, receipt paper, and so on. Widespread use of BPA-containing products leads to ubiquitous BPA exposure. Growing evidence indicates that environmental contaminants cause adverse health effects and poor developmental outcomes. BPA can affect the actions of endogenous hormones and disrupt endocrine function^1, 2^. Because of its toxic effect, some countries have banned BPA in feeding bottles for babies.

Research on the effect of BPA on reproduction is vast and has revealed altered hormone release^3, 4^, abnormal reproductive organs^5^. People are exposed to BPA mainly through skin contact and food ingestion. A study performed in Europe confirmed the presence of BPA in the urine of 97.7% of the participants^6^. BPA results in adverse reproductive outcomes. High BPA concentrations in urine are related to a decrease in ovarian response, number of fertilized oocytes, and blastocyst formation^7^.

The exposure of BPA to oocytes leads to the failure of maturation and occurrence of MII abnormalities, including spindle morphology, chromosome misalignment, and aberrant actin distribution^8, 9^. These abnormalities are associated with poor developmental outcomes. Affected embryos have shown decreased competency, increased apoptosis, and a skewed sex ratio^10^. In addition, exposure to BPA causes an adverse effect on sperm, such as a decrease in number and quality as well as DNA damage^11^. In animal models where BPA was administered orally or via injection, there were several abnormal reproductive effects, such as the early onset of puberty^12^ and development of reproductive tracts and organs^13^.

Several studies have shown that some cellular processes, including oxidative stress, DNA damage, and epigenetic modifications are essential for early embryonic development^14–16^. Many studies have demonstrated the negative effect of BPA exposure during early embryonic development^10, 17^. There is little research focused on the mechanism of action of BPA on porcine embryonic development. Therefore, the objective of this study was to evaluate the influence and mechanism of BPA on porcine early embryonic development, oxidative stress, DNA damage, apoptosis, autophagy, epigenetic modification, and inner cell mass formation.

## Results

### BPA exposure influences blastocyst formation during porcine early embryonic development

**Figure 1:**
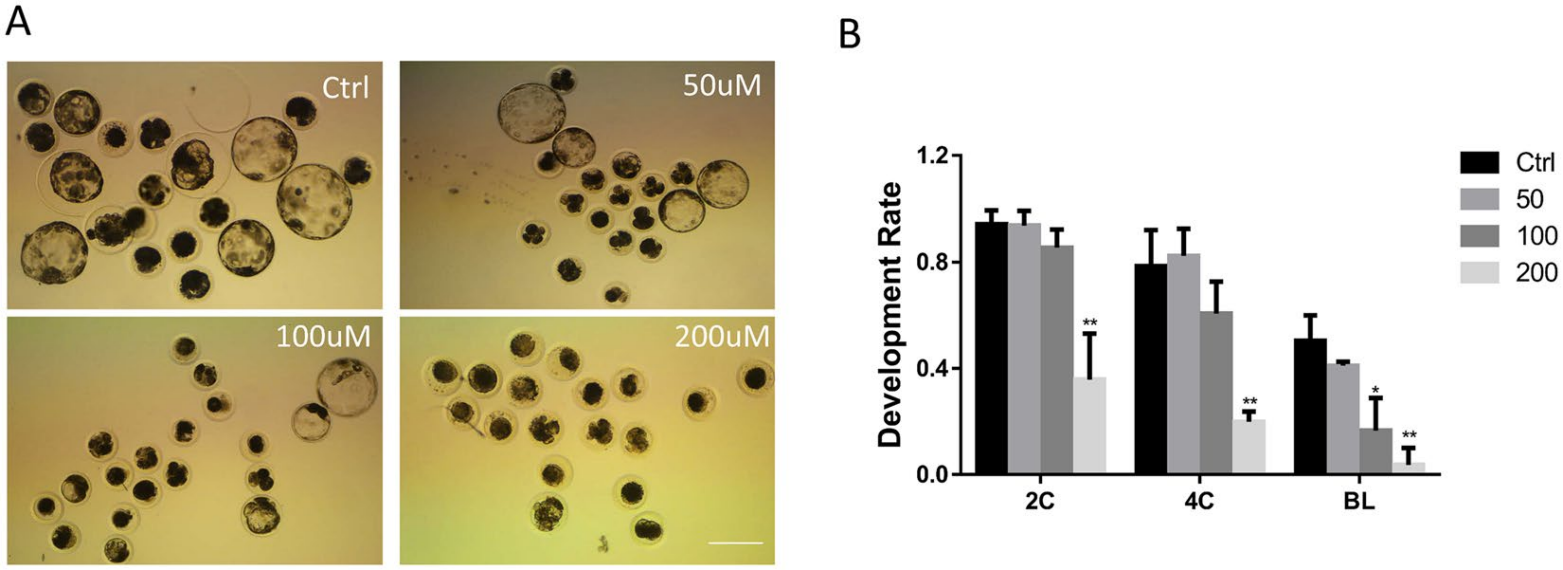
Effect of BPA on porcine early embryonic development. (**A**) Effect of different BPA concentrations on embryonic development. (**B**) The rate of embryonic development. *P < 0.05, **P < 0.01. Ctrl, control; Treat, treatment. 2 C: 2-cell; 4 C: 4-cell; BL: Blastocyst. Scale bar: 200 μm.

To detect the function of BPA on embryonic development, parthenotes were incubated in the *in vitro* culture (IVC) medium for 7 days in the presence of BPA at different doses (0, 50, 100, and 200 μM) and the blastocyst rates were examined. As shown in Fig. 1A,B, BPA exposure resulted in an effective decrease of blastocyst formation at concentrations of 100 and 200 μM (16.5 ± 7.1%, P < 0.05 and 3.7 ± 3.7%, P < 0.01), but not 50 μM (41.0 ± 1.0%) compared with the control (50.3 ± 5.5%). We also found that more treated parthenotes were arrested at the 4-cell stage. The proportion of parthenotes at the 2-cell stage with BPA treatment (50 and 100 μM) (93.7 ± 3.2% and 85.4 ± 3.9%) was not significantly different from that in the control group (94.1 ± 3%). However, there was a significant difference with 200 μM BPA (35.8 ± 9.9%, P < 0.01). Most of the parthenotes can develop to the 4-cell stage at concentrations of 0, 50, and 100 μM (78.4 ± 7.8%, 82.2 ± 7.2%, and 60.5 ± 7.0%, respectively), but not with 200 μM (19.8 ± 2.2%, P < 0.01). BPA concentration of 100 μM was used in all further experiments.

### BPA exposure results in reactive oxygen species (ROS) generation

**Figure 2:**
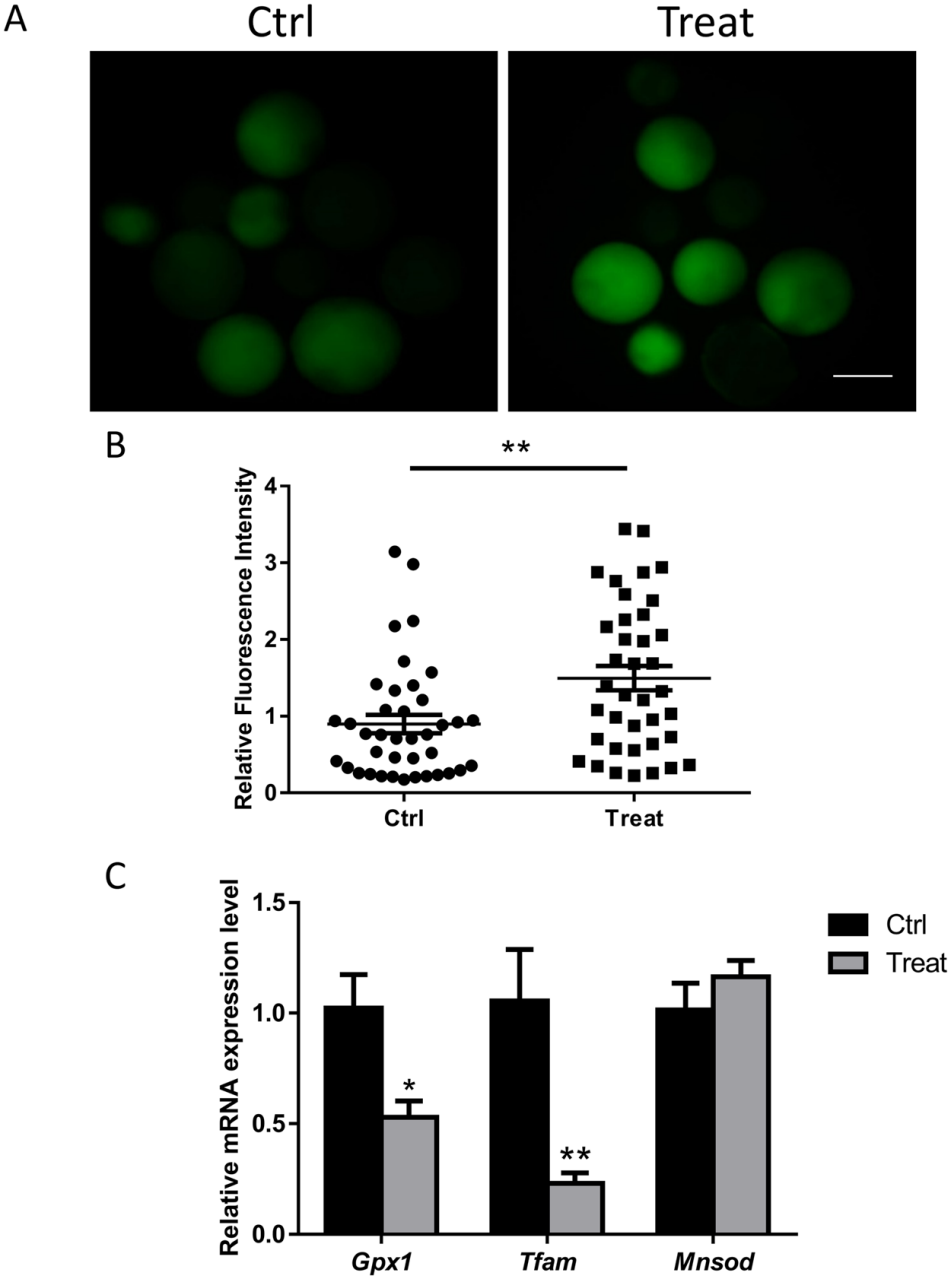
BPA increases ROS. (**A**) Staining for ROS in porcine blastocysts in the control and BPA-exposed groups. (**B**) Green fluorescence intensity was measured. (**C**) mRNA expression of *Gpx1*, *Mnsod*, and *Tfam* in blastocysts with or without BPA. **P < 0.01. Ctrl, control; Treat, treatment. Scale bar: 200 μm.

It has been demonstrated that ROS can be generated when exposed to environmental contaminants. Oxidative stress can also occur in a biological system when exposed to environmental contaminants and be defined as an imbalance between production and depletion of ROS^18^. Therefore, in this study, ROS levels were examined after BPA exposure. As shown in Fig. 2A, the generation of ROS increased in the treatment group compared with that in the control group. The relative green fluorescence intensity that induced ROS production was significantly increased (Fig. 2B). The mRNA expression level of oxidative stress-related genes (*Gpx1*, *Tfam*, and *Mnsod*) was also examined. The *Gpx1* and *Tfam* mRNA levels significantly decreased compared with that in the control group. The *Mnsod* expression level showed no significant variation (Fig. 2C).

### Effect of BPA exposure on apoptosis

**Figure 3:**
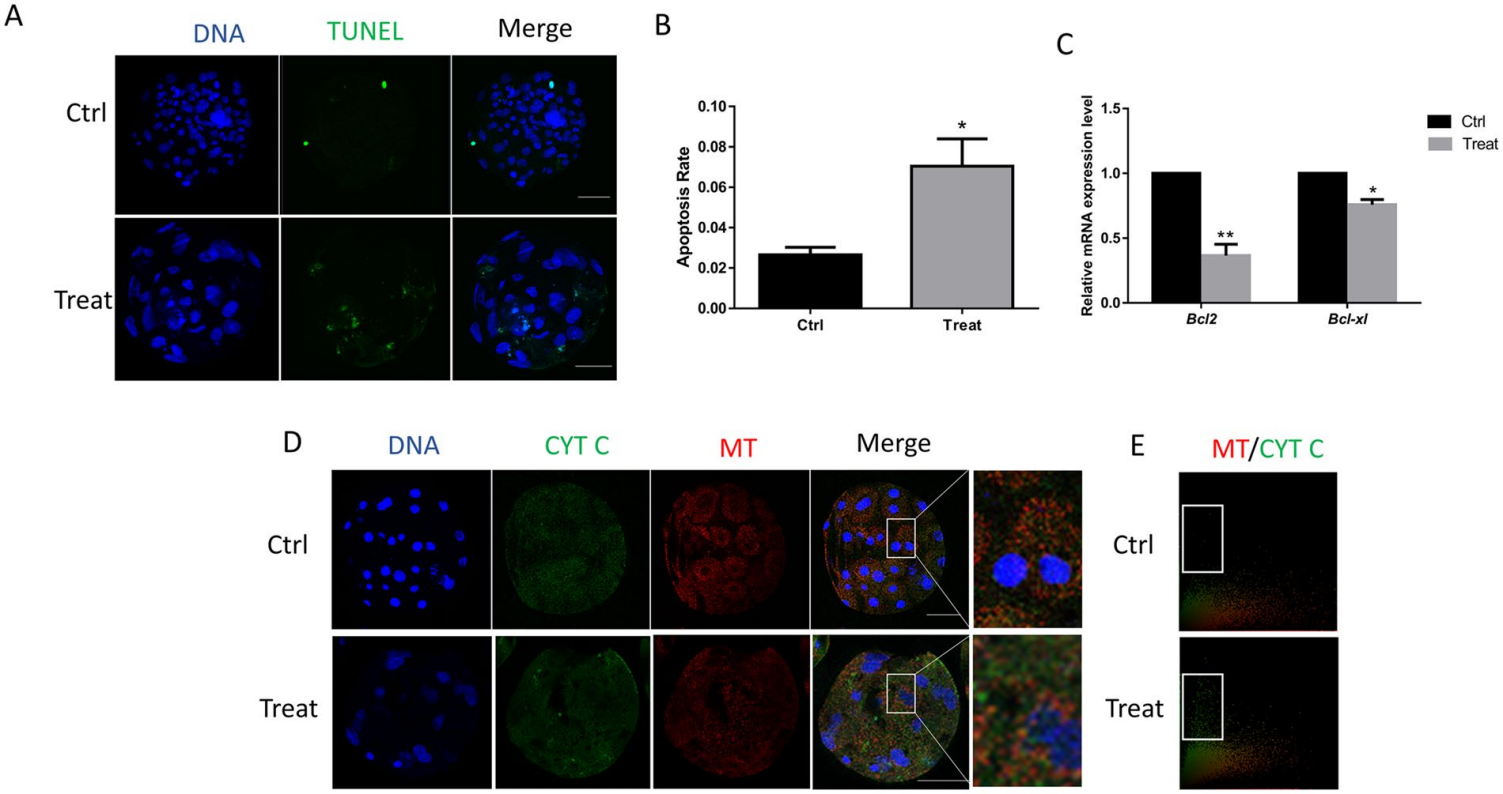
Effect of BPA exposure on apoptosis. (**A**) TUNEL-positive cells were detected in the presence or absence of BPA. (**B**) The rate of TUNEL-positive and total cells are shown. (**C**) Relative mRNA expression level of *Bcl2* and *Bcl-xl* in blastocysts with or without BPA exposure. (**D**) The localization of cytochrome c and mitochondria was detected using immunostaining. (**E**) The localization of cytochrome c and mitochondria in the whole blastocyst. *P < 0.05. Ctrl, control; Treat, treatment. Scale bar: 50 μm. MT: mitochondria; CYT C: cytochrome c.

Apoptosis was examined after BPA exposure by using the TUNEL assay. The apoptotic effect was calculated as the ratio between the number of TUNEL-positive nuclei and the total cell number. As shown in Fig. 3A, more TUNEL-positive nuclei were observed in the treatment group. The ratio of the TUNEL-positive nuclei significantly increased after BPA exposure (Fig. 3B). The expression of apoptosis-related genes was also analyzed after treatment with BPA. The expression of both anti-apoptotic genes, *Bcl2* and *Bcl-xl*, significantly reduced (Fig. 3C,D), suggesting that BPA exposure may induce apoptosis.

The release of cytochrome c from the mitochondria into the cytoplasm initiates apoptosis. The co-localization of cytochrome c and mitochondria was examined using immunofluorescence staining. The release of cytochrome c from the mitochondria is shown in Fig. 3D after exposure to BPA. As shown in Fig. 3E, the control group showed obvious co-localization of mitochondria and cytochrome c in the whole blastocyst compared with the treatment group. The spots inside of white box displayed the cytochrome c which was released from the mitochondria. More cytochrome c was released from the mitochondria in the treatment group. Therefore, BPA exposure caused the release of cytochrome c and induced the initiation of apoptosis.

### BPA exposure increases P53 and P21 expression in the blastocyst

**Figure 4:**
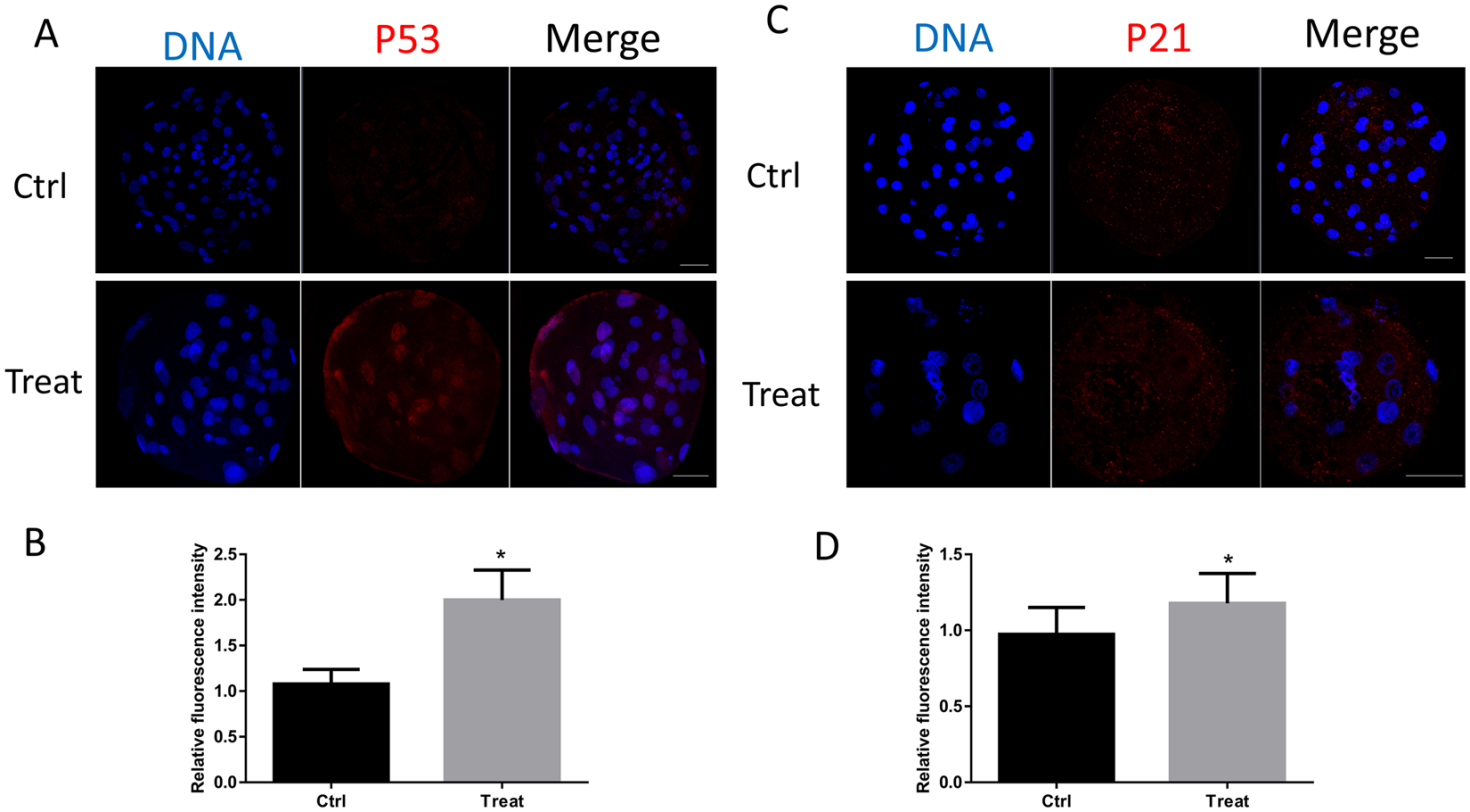
BPA treatment triggers the activation of the p53 pathway. (**A**) Staining for p53 showed its activation and expression. (**B**) The relative intensity of p53 is shown as bars. (**C**) Laser scanning confocal microscopy images of immunostaining for p21. (**D**) The relative intensity of p21 was measured. *P < 0.05. Ctrl, control; Treat, treatment. Scale bar: 50 μm.

In order to detect the mechanism of the negative effects of BPA on embryonic development, the activation of the p53 pathway was examined. The activation of p53 can result in poor developmental potential^19^. Therefore, we showed that BPA treatment could cause an increased expression of p53 (Fig. 4A,B). The expression of p21, a known downstream target of p53, was also examined. The fluorescence intensity of p21 increased after embryos were treated with BPA (Fig. 4C,D). These results show that BPA exposure impaired the preimplantation embryo development through the p53-p21 pathway.

### BPA exposure results in a decrease of pluripotency markers

**Figure 5:**
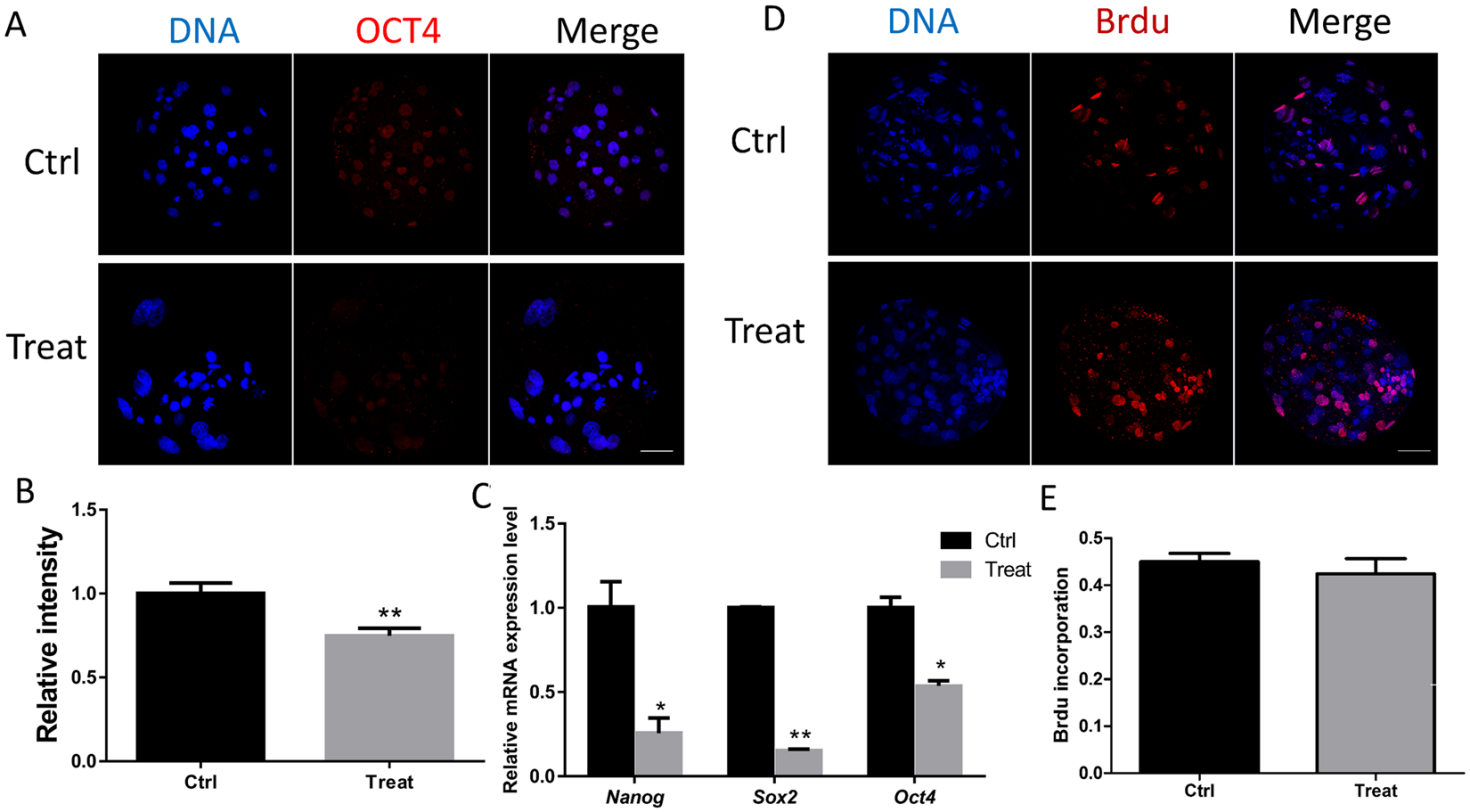
Effect of BPA treatment on pluripotency and differentiation. (**A**) Laser scanning confocal microscopy images of immunostaining for OCT4 in porcine blastocysts in the control and BPA treatment groups. (**B**) The relative intensity of OCT4 was measured. (**C**) The relative *Oct4*, *Nanog*, and *Sox2* mRNA expression levels are shown. (**D**) BrdU incorporation in blastocysts after BPA exposure. (**E**) Graph summarizing the relative rate of proliferation. *P < 0.05, **P < 0.01. Ctrl, control; Treat, treatment. Scale bar: 50 μm.

Embryonic development is associated with the embryo quality. As the key regulator of pluripotency, OCT4 expression was examined using immunofluorescence staining. The relative fluorescent density of OCT4 decreased in the BPA-exposed group compared with that in the control group (P < 0.05) (Fig. 5A,B). To confirm the effect of BPA on pluripotency, the expression of three pluripotent-related genes was examined. As shown in Fig. 5C, the mRNA expression level of *Oct4*, *Sox2*, and *Nanog* significantly reduced (P < 0.05), which is consistent with the results of immunofluorescence staining. Next, we detected the differentiation where there was no difference between the control and BPA-exposed groups (Fig. 5D,E). These results indicate that BPA exposure can decrease embryo quality.

### BPA exposure causes autophagy

**Figure 6:**
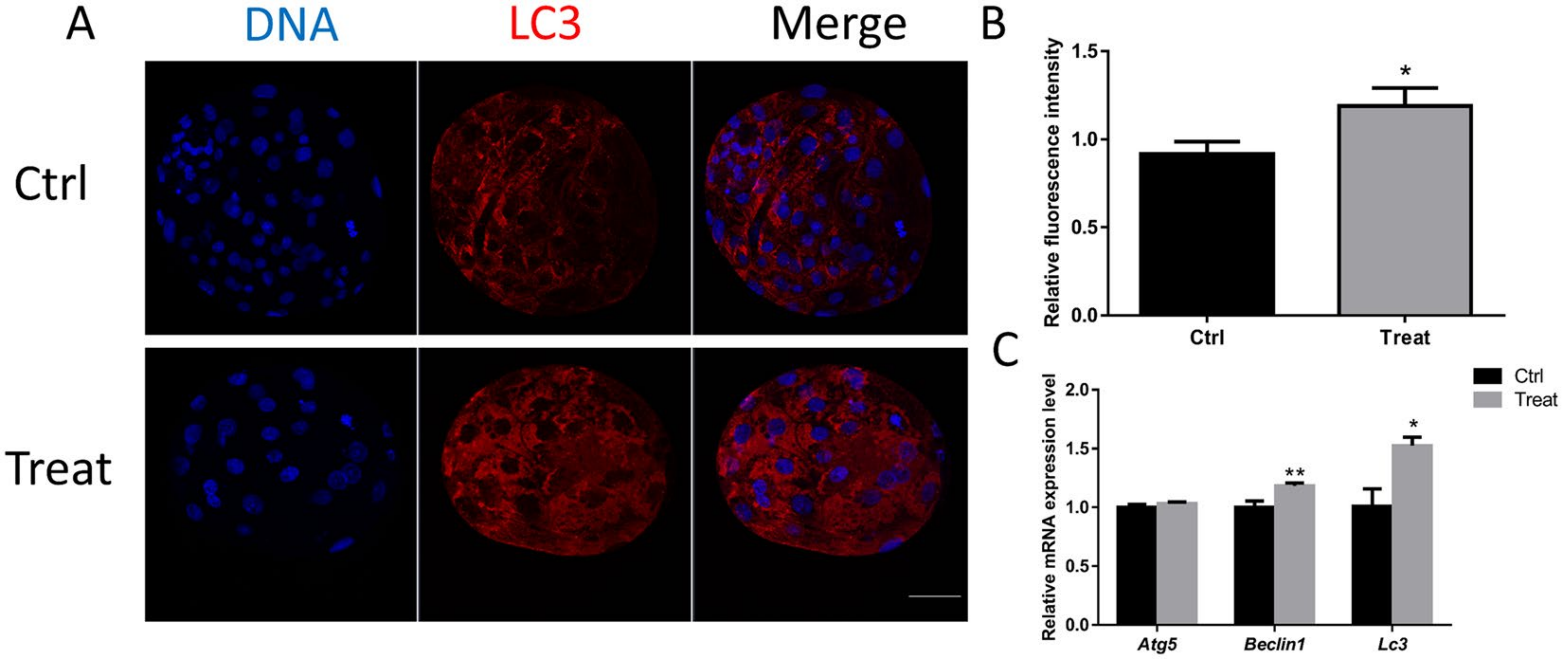
Effect of BPA exposure on autophagy. (**A**) Laser scanning confocal microscopy images of immunostaining for the LC3 protein in porcine blastocysts with or without BPA. (**B**) Relative fluorescence intensity of LC3. (**C**) Relative mRNA expression levels of autophagy-related genes, *Atg5*, *Beclin1*, and *Lc3*. *P < 0.05, **P < 0.01. Ctrl, control; Treat, treatment. Scale bar: 50 μm.

Oxidative stress can induce cell autophagy. Therefore, the expression level of LC3 was examined after treatment with BPA. Immunofluorescence staining showed a significant increase in fluorescence intensity (Fig. 6A,B). Consistent with this, the mRNA expression level of autophagy-related genes also significantly increased (Fig. 6C).

### BPA exposure causes the alternation of methylation

**Figure 7:**
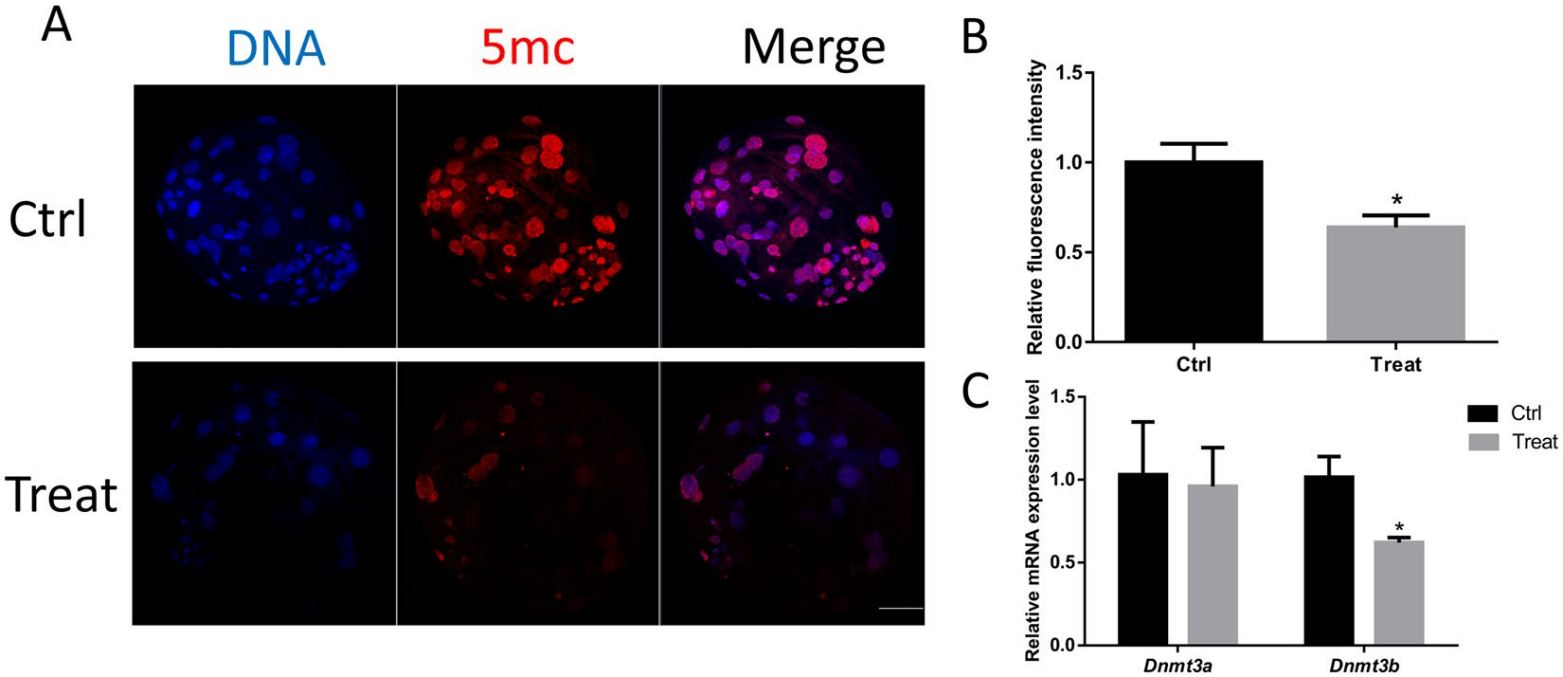
BPA exposure results in the alternations of methylation. (**A**) Immunostaining for 5 mC was detected between the control and BPA treatment groups. (**B**) Relative fluorescence intensity of 5 mC is shown as bars. *P < 0.05. Ctrl, control; Treat, treatment. Scale bar: 50 μm.

To evaluate the effect of embryo exposure to BPA on epigenetic modification, the level of 5-methyl-cytosine (5mC) was examined. As shown in Fig. 7A,B, we found that 5mC was co-localized with DNA and the relative intensity decreased compared with that in the control (P < 0.05). The mRNA expression of DNA methyltransferases (Dnmt3a and Dnmt3b) was analyzed using RT-PCR. mRNA levels were found to be significantly decreased after exposure to BPA (Fig. 7C).

## Discussion

People increasingly focus on the adverse effect of BPA because of its worldwide use and being detected in food and water consumed by people and animals. Accumulating evidence implicates that environmental toxicity impacts metabolism, the immune system, and reproduction. Several environmental factors that can perturb embryonic development have been reported. These factors have an effect on conditions or techniques used in assisted reproductive technologies (ART). Although the number of babies that are conceived through ART is growing, these individuals represent a relatively small percentage (1–4%) of babies born in industrialized countries^20^. Despite a number of studies have reported the toxic effect of BPA, the mechanism of BPA adverse effect is unknown. In this study, the mechanism of the negative effects of BPA on porcine early embryonic development was determined. Our results demonstrate that BPA treatment leads to the arrest of embryonic development at the 4-cell stage. In addition, the mechanism of BPA toxicity was explored by assessing DNA damage, apoptosis, autophagy, and epigenetic modification.

Previously, some studies have shown that BPA elicits its function through estrogen-mediated pathways by binding estrogen receptors, therefore, regulating gene expression. Sometimes, BPA shows a dual effect, which can exert pro- and anti-estrogenic effects^21, 22^. In the present study, we found that BPA causes the embryonic development arrest. Other previous studies have demonstrated that BPA reduced blastocyst development and metabolism in bovine and mouse embryos^17, 23^. The adverse effect of BPA exposure during oocyte maturation leads to alterations in subsequent embryonic development^10^. Most studies have focused on the function of BPA and regarded it as a xenoestrogen; therefore, the mechanism of BPA is via the estrogen-mediated effect.

In this study, we found that BPA modulates oxidative stress in porcine embryonic development. *In vitro* produced embryo displays low quality and small scale production. The major obstacles in *in vitro* embryo development are the production of excessive free radicals and exposure to oxidative stress. When ROS production exceeds the antioxidant capacity of embryos, oxidative stress occurs^24, 25^. The production of ROS is particular critical in early embryonic development, and excessive ROS will induce apoptosis and metabolic disorders^26, 27^. Several studies have implicated a close relationship between BPA toxicity and the generation of ROS, which results in oxidative stress in tissues^28, 29^. We also showed that embryos suffered oxidative stress, which was demonstrated by the increased production of ROS and oxidative stress-related gene mRNA expression of *Gpx1* and *Tfam*. *Gpx1* plays a role in the detoxification of hydrogen peroxide, and *Tfam* can stabilize mitochondria and thus affects oxidative stress^30, 31^.

Excessive ROS production leads to different kinds of injuries including mitochondria. In the present study, we observed that BPA causes the generation of ROS. It also resulted in mitochondrial damage, as observed by the release of cytochrome c from mitochondria. Cytochrome c is typically localized between the inner and outer membranes of mitochondria. However, during apoptosis, it is released into the cytoplasm, where it binds to apoptotic protease activating factor 1 (APAF1). The release of cytochrome c from mitochondria is, therefore, an important event in apoptosis initiation^32^. The apoptosis related genes (*Bcl-2*, *Bcl-xl*) mRNA expression confirms the effect of BPA on apoptosis The anti-apoptotic *Bcl-2* members prevent mitochondrial protein release^33, 34^. The result of *Bcl2* and *Bcl-xl* mRNA expression analysis confirmed the effect of BPA on apoptosis.

BPA also can cause DNA damage, which may occur via BPA-induce ROS production in porcine parthenotes^35, 36^. However, the effect of BPA exposure on DNA damage has not been extensively investigated. In this study, we found that the possible mechanism of BPA-induce DNA damage may be from the p53 pathway. DNA damage-induced phosphorylation of p53 results in the growth suppression. An increase in p53 and p21 levels were observed in BPA-treated embryos. These results suggest that BPA exposure results in DNA damage through oxidative stress.

As described above, BPA induced an increase in ROS levels, which leads to detrimental effects, such as mitochondrial and DNA damage. Generation of ROS is critical for cell survival and death, as well as autophagy. Among the various molecular components and signaling cascades related to autophagy induction, cellular redox status has been regarded as a critical mediator or regulator of autophagy^37, 38^. Our results implicate that BPA-induced autophagy, which was demonstrated by the increased level of LC3 and alteration of the mRNA expression of autophagy-related genes. Autophagy is a cellular mechanism, through which cells digest organelles, part of the cytoplasm, or proteins to circumvent nutrient deprivation or accumulation of damaged proteins/organelles. The importance of autophagy has mostly been attributed to its ability to remove damaged organelles and altered proteins resulting from programmed cell death^39–41^. Autophagy regulation and development are closely related to cell death machinery, especially in the regulation of apoptosis. In this study, BPA exposure led to mitochondrial and DNA damage, this causes autophagy as a protective effect.

Accumulating evidence indicates that BPA treatment may alter the epigenome, including DNA methylation and histone modification. Previous studies have suggested that BPA treatment disrupts the methylation status^22, 42^. The reduced expression of 5mC and *Dnmt* genes observed in the present study was caused by BPA exposure. DNA methylation is modulated by DNMTs, which results in the formation of 5mC. DNA methylation can correlate with gene transcription.

The embryonic developmental potential is influenced by pluripotency^43^. In addition, excessive DNA damage can induce apoptosis and decrease pluripotency-related gene expression in pigs^44^. In this study, the reduced expression of pluripotency-related genes might be induced by DNA damage and apoptosis.

**Figure 8:**
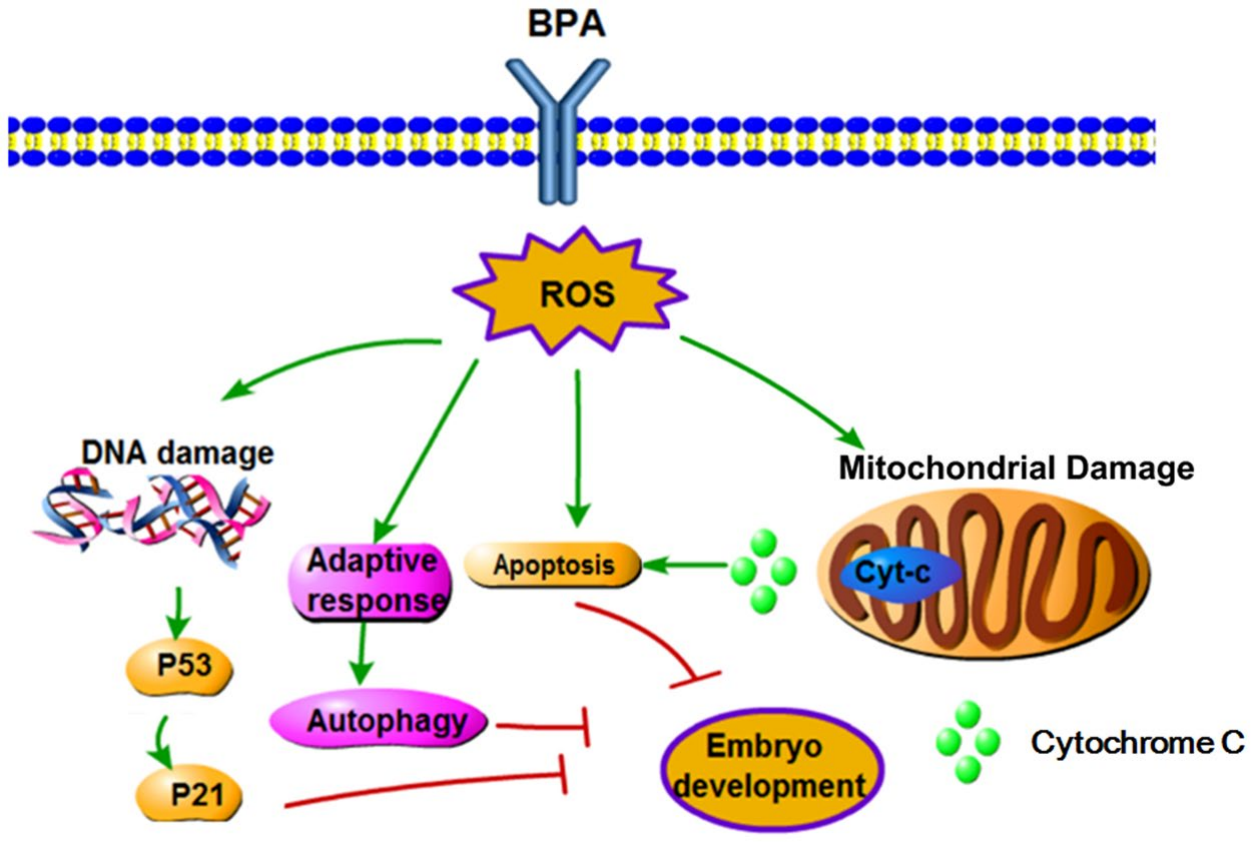
Schematic representation of the adverse effect of BPA during porcine early embryonic development. BPA exposure causes the generation of oxidative stress, which results in DNA and mitochondrial damage. Apoptosis was induced by the release of cytochrome c from the mitochondria because of mitochondrial damage. The DNA damage led to the activation of the p53-p21 pathway. In addition, the oxidative stress induced the adaptive response, autophagy.

In conclusion, the toxic effect of BPA on porcine embryonic development was confirmed in this study. BPA exposure to embryos caused increased levels of ROS resulting in oxidative stress. The oxidative stress caused mitochondrial and DNA damage, which leads to autophagy. In addition, BPA exposure can result in the alteration of DNA methylation, which may affect the health of offspring (Fig. 8).

## Materials and Methods

All chemicals used in this study were purchased from Sigma-Aldrich (St. Louis, MO, USA) unless otherwise indicated.

### Oocyte collection, *in vitro* maturation, and embryo culture

Ovaries from prepubertal gilts were obtained from a local slaughterhouse, maintained in saline at 37 °C, and transported to the laboratory. Follicles that were 3–6 mm in diameter were aspirated. Cumulus-oocyte complexes (COCs) that were surrounded by more than three layers of cumulus cells were selected for culture. COCs were isolated from follicles and washed three times with TL-HEPES. COCs were cultured in tissue culture medium 199 (TCM 199) with 10% porcine follicular fluid, 0.1 g/L sodium pyruvate, 0.6 mM L-cysteine, 10 ng/mL epidermal growth factor, 10 IU/mL luteinizing hormone, and 10 IU/mL follicle stimulating hormone at 38.5 °C for 44 h in a humidified atmosphere of 5% CO_2_.

After maturation, cumulus cells were removed with 0.1% hyaluronidase and repeated pipetting. For activation of parthenogenesis, oocytes with polar bodies were selected. They were activated by two direct current pulses of 1.1 kV/cm for 60 µs and then incubated in porcine zygote medium (PZM-5) containing 7.5 μg/mL of cytochalasin B for 3 h. Finally, approximately 90 embryos were cultured in PZM-5 for 8 days at 38.5 °C in a humidified atmosphere of 5% CO_2_. On the fifth day, 10% fetal bovine serum was added to the medium. To determine the effect of BPA on early porcine embryonic development, BPA was added to the medium after activation at final concentrations of 100 or 200 μM. The 100 μM concentration was used in the following experiments as it represents the minimum concentration that induces an effect on blastocyst formation.

### Reactive oxygen species (ROS) staining

Blastocysts (n = 39, 3 replicates) were incubated for 15 min in IVC medium containing 10 µM 2′,7′-dichlorodihydrofluorescein diacetate (H_2_DCF-DA) at 37 °C. After incubation, blastocysts were washed three times with IVC medium and transferred to PBS drops covered with paraffin oil in a polystyrene culture dish. The fluorescent signal was captured using an epifluorescence microscope (Nikon Corp., Tokyo, Japan). The fluorescence intensity in the control group was arbitrarily set at 1, and the fluorescence intensities in the treatment groups were then measured and expressed as relative values for the control group.

### Terminal deoxynucleotidyl transferase-mediated 2′-deoxyuridine 5′-triphosphate (dUTP) nick-end labeling (TUNEL) assay

After the embryos had been treated with BPA, the blastocysts were collected. The blastocysts (n = 36, 3 replicates) were then fixed in 3.7% paraformaldehyde for 15 min at room temperature and subsequently permeabilized by incubation in 0.5% Triton X-100 for 30 min at 37 °C. The embryos were incubated with fluorescein-conjugated dUTP and the terminal deoxynucleotidyl transferase enzyme (*In Situ* Cell Death Detection Kit, Roche; Mannheim, Germany) for 1 h at 37 °C, and then washed three times with PBS/PVA. Embryos were treated with Hoechst 33342 for 5 min, washed three times with PBS/PVA, and mounted onto glass slides. Images were captured using a confocal microscope (Zeiss LSM 710 META, Jena, Germany).

### Immunofluorescence and confocal microscopy

Embryos (n = 38, 3 replicates) were fixed in 3.7% paraformaldehyde for 20 min at room temperature, permeabilized with PBS/PVA containing 0.5% Triton X-100 at 37 °C for 1 h, and then incubated in PBS/PVA containing 1.0% bovine serum albumin at 37 °C for 1 h. Subsequently, the embryos were incubated overnight at 4 °C with anti-LC3 (ab58610, 1:100; Abcam, Cambridge, UK), anti-cytochrome C (ab110325, 1:100; Abcam), anti-p53 (sc6243, 1:100; Santa Cruz Biotech, CA, USA) and anti-OCT4 (sc8628, 1:100; Santa Cruz Biotech) antibodies. To check the fluorescence signal of 5mC, blastocysts were denatured with 1 N HCl at room temperature for 30 min and neutralized with 0.1 M Tris-HCl, pH 8.0 for 15 min. Subsequently, blastocysts were incubated in PBS containing 1% BSA, and then incubated overnight at 4 °C with 5mC antibody (ab10805, 1:100, Abcam, Cambridge, UK). After washing three times with PBS/PVA, the oocytes and embryos were incubated at 37 °C for 1 h with either goat anti-rabbit IgG (A11011, 1:200, Invitrogen) or rabbit anti-goat IgG (A11079, 1:200, Invitrogen), anti-mouse IgG (A21202, 1:200, Invitrogen). The oocytes and embryos were then stained with Hoechst 33342 for 5 min, washed three times with PBS/PVA, mounted onto slides, and examined using a confocal microscope (Zeiss LSM 710 META, Jena, Germany). Images were processed using Zen software (version 8.0, Zeiss, Jena, Germany).

### 5-Bromo-deoxyuridine analysis

The rate of cell proliferation was analyzed using 5-bromo-deoxyuridine (BrdU). Briefly, day 7 blastocysts (n = 32, 3 replicates) were incubated with 100 μM BrdU for 6 h at room temperature and then washed three times with PBS/PVA. Blastocysts were fixed in 3.7% paraformaldehyde for 20 min, and then permeabilized with PBS/PVA containing 0.5% Triton X-100 for 30 min. They were treated with 1 N HCl at room temperature for 30 min. Blastocysts were incubated in PBS/PVA containing 3.0% bovine serum albumin at 37 °C for 1 h and then with anti-BrdU at 4 °C overnight. After washing with PBS/PVA three times, blastocysts were incubated at 37 °C for 1 h with goat anti-mouse antibody. Finally, blastocysts were stained with Hoechst 33342 for 5 min, mounted onto slides, and examined using a confocal microscope.

### Real-time reverse transcriptase-polymerase chain reaction (RT-PCR)

Day 8 blastocysts were collected and mRNA was extracted from 10 blastocysts per group using a DynaBeads mRNA Direct Kit (Dynal Asa, Oslo, Norway), according to the manufacturer’s instructions. cDNA was obtained via reverse transcription of mRNA using the Oligo (dT)12–18 primer and SuperScript III Reverse Transcriptase (Invitrogen Co., Grand Island, NY, USA). The amplification cycles were as follows: 95 °C for 3 min followed by 40 cycles of 95 °C for 15 s, 60 °C for 30 s, and 72 °C for 20 s, with a final extension at 72 °C for 5 min. Relative gene expression was normalized to internal porcine *Gapdh* mRNA levels using the 2^−ΔΔCt^ method

### Statistical analysis

All data were analyzed using the one-way analysis of variance (ANOVA) and Chi-square test implanted in Statistical Package for the Social Sciences (SPSS). Differences among treatments were examined using the Duncan multiple range test. Data were expressed as mean ± standard error of the mean. Each experiment was performed in triplicate and differences were considered significant if P < 0.05.
